# Supplementary material for: An open-source framework for large-scale, flexible evaluation of biomedical text mining systems
Source: J Biomed Discov Collab. 2008 Jan 29;3:1. doi: 10.1186/1747-5333-3-1 (PMC2276192; doi:10.1186/1747-5333-3-1)
Supplement: Additional file 1 — Evaluation platform source code. The additional file contains the software evaluation framework discussed in this paper. The most current release can be downloaded from SourceForge.net [1]. See the accompanying README file for instructions on installation and use. Also included in the distribution is a collection of UIMA wrappers for some commonly used BioNLP tools and annotated corpora. [file 1747-5333-3-1-S1.zip › bionlp_uima_repository-1.0.1/docs/api/src-html/edu/uchsc/ccp/uima/ae/nlp/opendmap/OpenDmapAnnotator.html]

```
001    /**
002     * The OpenDMAP for Protege Project
003     * October 2005
004     */
005    package edu.uchsc.ccp.uima.ae.nlp.opendmap;
006    
007    import java.util.ArrayList;
008    import java.util.Collection;
009    import java.util.Vector;
010    import java.util.logging.Level;
011    
012    import org.apache.uima.analysis_engine.ResultSpecification;
013    import org.apache.uima.analysis_engine.annotator.AnnotatorContext;
014    import org.apache.uima.analysis_engine.annotator.AnnotatorContextException;
015    import org.apache.uima.analysis_engine.annotator.AnnotatorInitializationException;
016    import org.apache.uima.analysis_engine.annotator.AnnotatorProcessException;
017    import org.apache.uima.analysis_engine.annotator.JTextAnnotator_ImplBase;
018    import org.apache.uima.jcas.JCas;
019    
020    import edu.stanford.smi.protege.model.KnowledgeBase;
021    import edu.stanford.smi.protege.model.Project;
022    import edu.uchsc.ccp.opendmap.Parser;
023    import edu.uchsc.ccp.opendmap.configuration.Configuration;
024    import edu.uchsc.ccp.opendmap.configuration.ConfigurationException;
025    import edu.uchsc.ccp.opendmap.pattern.ParseException;
026    
027    /**
028     * This class is a wrapper for OpenDMAP to help fit it into the
029     * UIMA framework.
030     * <p>
031     * Right now this class hides many of the details involved in
032     * setting up the parser, connecting it to Protege, and loading
033     * pattern definitions.
034     * <p>
035     * To build a UIMA annotator with OpenDMAP, extend this class.
036     * 
037     * @author R. James Firby
038     */
039    public abstract class OpenDmapAnnotator extends JTextAnnotator_ImplBase {
040    
041            /* The parser instance to use in the UIMA pipeline. */
042            private Parser parser = null;
043            
044            /* The Protege project and pattern files to load into the parser */
045            private String protegeProjectFile = null;
046            private Vector<String> patternFiles = null;
047            
048            /**
049             * Create a new, empty OpenDMAP Annotator.
050             */
051            public OpenDmapAnnotator() {
052                    super();
053                    protegeProjectFile = null;
054                    patternFiles = new Vector<String>();
055            }
056            
057            /**
058             * Set the Protege Project file to be loaded into the
059             * OpenDMAP parser when this annotator is initialized.
060             * Only one project file may be used for each annotator.
061             * 
062             * @param filename The name of the Protege project file
063             */
064            public void setProtegeProjectFilename(String filename) {
065                    protegeProjectFile = filename;
066            }
067            
068            /**
069             * Add the name of an OpenDMAP pattern file to be loaded
070             * into the parser when this annotator is initialized.
071             * Any number of pattern files may be added.
072             * 
073             * @param filename The name of one pattern file
074             */
075            public void addPatternFilename(String filename) {
076                    patternFiles.add(filename);
077            }
078            
079            /**
080             * Initialize the OpenDMAP parser and load the Protege
081             * project and the pattern files.
082             */
083            public void initialize(AnnotatorContext context) throws AnnotatorInitializationException {
084                    // Create a parser that ignores case (for ease of typing)
085                    //  and generates tracing output (if desired).
086                    parser = new Parser(false, Level.OFF);
087                    
088                    // If a configuration file is specified, load it
089                    String configurationFile = null;
090                    try {
091                            // Get the configuration filename from the UIMA context
092                            Object configFile = context.getConfigParameterValue("configurationFile");
093                            if (configFile instanceof String)
094                                    configurationFile = (String) configFile;
095                    } catch (AnnotatorContextException e) {
096                            // Cannot access annotator configuration
097                            throw new AnnotatorInitializationException("Cannot get OpenDMAP configuration file name from UIMA context", new Object[]{this}, e);
098                    }
099                    if (configurationFile != null) {
100                            // Configure the parser using the configuration file
101                            try {
102                                    Configuration configurator = new Configuration(configurationFile);
103                                    configurator.configure(parser);
104                            } catch (ConfigurationException e) {
105                                    // Can't process configuration
106                                    throw new AnnotatorInitializationException("Cannot initialize OpenDMAP from configuration file '" + configurationFile + "'", new Object[]{this});
107                            }
108                    }
109                    
110                    // Add recognition patterns to the parser
111                    if (!patternFiles.isEmpty()) {
112                            Collection errors = new ArrayList();
113                            Project project = new Project(protegeProjectFile, errors);
114                            if (errors.size() != 0) {
115                                    // Throw the first error if one encountered
116                                    Object e = errors.iterator().next();
117                                    if (e instanceof Throwable) {
118                                            throw new AnnotatorInitializationException("Creating Protege project '" + protegeProjectFile + "'", new Object[]{this}, (Throwable) e);
119                                    } else {
120                                            throw new AnnotatorInitializationException("Creating Protege project '" + protegeProjectFile + "'", e.toString(), new Object[]{this});                                  
121                                    }
122                            } else {
123                                    // Load the pattern files
124                                    KnowledgeBase kb = project.getKnowledgeBase();
125                                    for (String patternFile: patternFiles) {
126                                            try {
127                                                    parser.addPatternsFromFile(patternFile, kb);
128                                            } catch (ParseException e) {
129                                                    // Throw an error if one occurs
130                                                    throw new AnnotatorInitializationException("Loading pattern file '" + patternFile + "'", new Object[]{this}, e);
131                                            }
132                                    }
133                            }
134                    }
135                    
136                    // At this point, the parser is ready to go
137            }
138    
139            /**
140             * When the UIMA pipeline signals it is time to run this
141             * annotator, call user code to get tokens from the jcas,
142             * call the parser, and then write annotations back to the
143             * jcas.
144             */
145            public void process(JCas jcas, ResultSpecification resultSpec) throws AnnotatorProcessException {
146                    // Reset the parser
147                    parser.reset();
148                    // Do the real work
149                    process(parser, jcas, resultSpec);
150            }
151            
152            /**
153             * Process the JCas as part of a UIMA pipeline.  Ie. get input
154             * tokens for OpenDMAP from the JCas, run the OpenDMAP parser,
155             * process the results and write appropriate annotations back
156             * to the JCas.
157             * 
158             * @param parser The OpenDMAP parser to use for parsing.
159             * @param jcas The JCas holding input tokens and output annotations.
160             * @param resultSpec The type of result expected to be put into the JCas.
161             */
162            public abstract void process(Parser parser, JCas jcas, ResultSpecification resultSpec);
163    
164    }
```
